# Ecological structure of diversity-dependent diversification in Phanerozoic marine bivalves

**DOI:** 10.1098/rsbl.2023.0475

**Published:** 2024-01-17

**Authors:** Michael Foote, Stewart M. Edie, David Jablonski

**Affiliations:** ^1^ Department of the Geophysical Sciences, University of Chicago, Chicago, IL 60637, USA; ^2^ Department of Paleobiology, National Museum of Natural History, Smithsonian Institution, Washington, DC 20560, USA

**Keywords:** diversity dynamics, diversity-dependence, macroevolution, Bivalvia, palaeoecology

## Abstract

Rigorous analysis of diversity-dependence—the hypothesis that the rate of proliferation of new species is inversely related to standing diversity—requires consideration of the ecology of the organisms in question. Differences between infaunal marine bivalves (living entirely within the sediment) and epifaunal forms (living partially or completely above the sediment–water interface) predict that these major ecological groups should have different diversity dynamics: epifaunal species may compete more intensely for space and be more susceptible to predation and physical disturbance. By comparing detrended standing diversity with rates of diversification, origination, and extinction in this exceptional fossil record, we find that epifaunal bivalves experienced significant, negative diversity-dependence in origination and net diversification, whereas infaunal forms show little appreciable relationship between diversity and evolutionary rates. This macroevolutionary contrast is robust to the time span over which dynamics are analysed, whether mass-extinction rebounds are included in the analysis, the treatment of stratigraphic ranges that are not maximally resolved, and the details of detrending. We also find that diversity-dependence persists over hundreds of millions of years, even though diversity itself rises nearly exponentially, belying the notion that diversity-dependence must imply equilibrial diversity dynamics.

## 1. Introduction

### (a) Diversity-dependent diversification in the history of life

Whether the rate of proliferation of new species depends on standing diversity remains a fundamental, largely unanswered question in ecology, evolutionary biology and palaeontology [1]. The fossil record of marine life often shows rates of diversification to be negatively correlated with diversity [1–7]. Periods of relatively high diversity in the oceans saw suppressed diversification rates compared to times when there were fewer taxa, as in the immediate aftermaths of mass extinctions. Despite abundant palaeontological evidence for diversity-dependence, this crucial issue remains contentious [1,8,9]. Long-term dynamics over tens to hundreds of millions of years are mostly understood from analyses of the global biota and a few major clades, and while the patterns are strong [2,7,10], the broad taxonomic scope of most analyses often encompasses ecologically disparate taxa that are unlikely to interact. Thoroughly testing potential drivers requires an analytical design that explicitly incorporates ecological differences among lineages and larger clades [5], focuses on species that have the potential to interact ecologically, draws data from clades with a robust and temporally well-resolved fossil record, and covers the long spans of time relevant to macroevolutionary dynamics. Marine bivalves satisfy these desiderata. Moreover, compared to analyses of only living taxa, long-term palaeontological time series can better delimit the roles of speciation and extinction [6,7,11,12].

### (b) Potential role for ecology in bivalve diversification

Competition for available space has been widely documented in living marine species [13,14] and has been argued to underpin diversity dynamics at the macroevolutionary scale in certain habitats [4,15–17]. Using the fossil record of marine bivalves, a model system for studying macroevolution through the Phanerozoic [3,18–20], we test for differences in the diversity-dependence of evolutionary rates in two principal life modes: *infaunal* forms, those living enclosed within a substratum, and *epifaunal* forms, operationally defined here to include those living entirely atop, attached to, or partly within the substratum [21–26].

Epifauna experience greater exposure to predation and substratum disturbance than infauna, both from physical processes (wave energy) and churning of sediments by other animals (bioturbation), the latter having substantially increased through the Phanerozoic [27–29]. Predation and bioturbation can limit the relative amounts of habitat available to epifauna, restricting those taxa to spatially limited refugia, hardgrounds or undisturbed soft sediment [27,30]. Thus, we would expect epifauna to show stronger negative diversity-dependence than infauna. Given the wide availability of infaunal habitats across continental shelves through time, we expect weaker diversity-dependence in the evolutionary rates of infauna, although, on ecological scales, certain infaunal bivalves show density-dependent migration or repositioning in response to crowding from other bivalve taxa [31,32]. Finally, we test whether the two ecological groups may conceivably interact as a coupled system; bioturbation by infauna might exclude epifauna from potential habitat [27,28,33], raising the possibility that elevated diversity and abundance of infauna negatively impact the evolutionary rates of the epifauna. At the same time, epifauna could potentially affect infauna by increasing the volume of coarse skeletal material in the sediment, thereby inhibiting infaunal burrowing [34,35].

## 2. Material and methods

### (a) Data

We analyse diversity dynamics using a database of 3365 fossil marine bivalve genera, compiled from a compendium of first and last stratigraphic occurrences [36] that has been heavily vetted and substantially expanded using the primary literature and museum collections over more than 20 years [18–20,23,37–39]. Genera were classified as infaunal (*N* = 2098) and epifaunal (*N* = 1267) using aspects of their functional morphology and phylogenetic affinity [21,22,24–26] (electronic supplementary material, tables S1,S2).

### (b) Analysis of diversity dynamics

Estimates of richness and taxonomic rates of evolution used the standard ‘boundary-crosser’ methods for stratigraphic range data [40]. To minimize edge effects [40,41] and the impact of sparse Cambrian and earliest Ordovician data, we analyse data from the Ordovician Floian Stage through the Miocene Epoch (electronic supplementary material, text; electronic supplementary material, table S1). This approach tacitly assumes that palaeontological completeness is high enough to treat observed first and last appearances as proxies for times of origination and extinction. Many methods exist for estimating rates with incomplete sampling [7,41,42], but the high fidelity of the bivalve record and the similar preservation potential of the two ecological groups obviate the need for complex approaches. In the spatial and temporal parts of the geological record analysed here, using methods in [43–47], we estimate that over 90% of bivalve genera are sampled at least once during their lifetimes, and, on average, over 90% of their original durations are represented by their preserved stratigraphic ranges (electronic supplementary material, table S3). These exceptionally high completeness estimates, even compared with those from an early version of this database [48], exceed estimates with data from the Paleobiology Database [49] (electronic supplementary material, table S3). High completeness reflects both the intrinsic preservability of bivalve molluscs [50] and the continued growth and vetting of the database, and it gives us confidence in inferring evolutionary dynamics.

**Figure 1.**
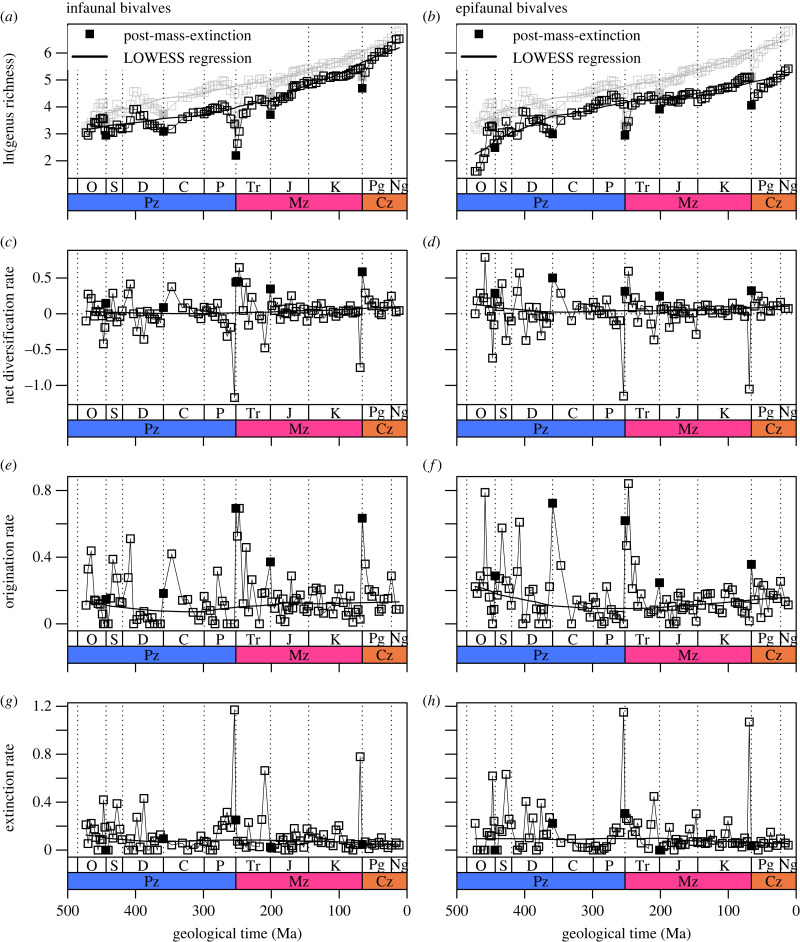
History of diversity and evolutionary rates. Grey: combined infauna + epifauna. Pz, Palaeozoic Era; Mz, Mesozoic Era; Cz, Cenozoic Era; O, Ordovician; S, Silurian; D, Devonian; C, Carboniferous; P, Permian; Tr, Triassic; J, Jurassic; K, Cretaceous; Pg, Palaeogene; Ng, Neogene; Ma, million years before present. Post-mass-extinction intervals: Lower Llandovery, Tournaisian, Induan, Hettangian, Danian.

**Figure 2.**
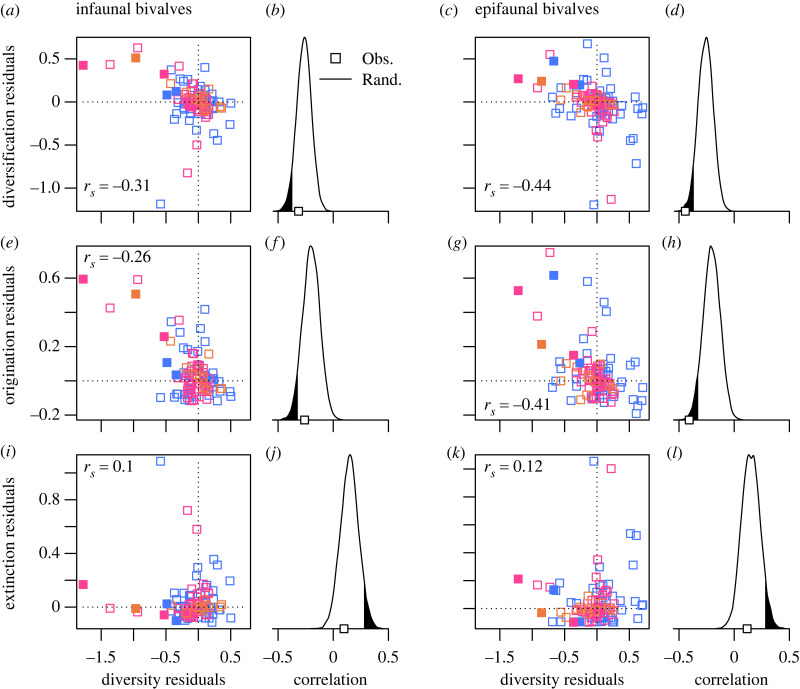
Correlations between diversity residuals and rate residuals. (*a*,*c*,*e*,*g*,*i*,*k*) Scatterplots of residuals, with rank-order correlations indicated. Colour coding of points corresponds to era-level designations of figure 1. (*b*,*d*,*f*,*h*,*j*,*l*) Comparison of observed correlations (open squares) between diversity residuals and rate residuals with correlations resulting from a randomization protocol (solid lines). Shaded area: one-sided 5% of randomized distribution.

Our approach to testing for diversity-dependent diversification follows recent studies [6,7,51] that compare standing diversity at the start of a time interval to rates of origination, extinction, and net diversification (origination minus extinction) in the ensuing part of that interval. To reflect the multiplicative nature of diversification, richness is expressed logarithmically. To avoid the assumption of a constant carrying capacity or a particular model of diversification [2,4,52,53], we detrend all time series, via LOWESS regression with a smoothing span *f* = 0.5, and measure the rank-order correlations between residuals of diversity and taxonomic rates relative to long-term trends (figures 1 and 2, electronic supplementary material, S1, S2; table 1). The hypothesis of diversity-dependence predicts a negative correlation between diversity residuals and diversification-rate residuals. Given the general statistical phenomenon of regression to the mean, diversity and diversification rate tend be negatively correlated even if rates are independent of diversity [54,55]. We therefore use a randomization procedure [6,7] to determine whether observed correlations are stronger than would be expected for a diversity-independent process.

**Table 1.** Spearman rank-order correlations (*r*_s_) between diversity residuals and rate residuals.

ecological group	diversification		origination		extinction	
	*r*_s_	*p^a^*	*r*_s_	*p^a^*	*r*_s_	*p^a^*
infauna + epifauna	−0.379	0.043	−0.348	0.031	0.016	0.926
infauna	−0.314	0.227	−0.260	0.218	0.097	0.734
epifauna	−0.443	0.004	−0.407	0.004	0.117	0.662

^a^Nominal one-tailed *p*-value is the proportion of randomizations that yield a correlation more extreme than the observed value.

All analyses were carried out in R version 4.3.0 [56].

## 3. Results and discussion

### (a) Contrasting diversification dynamics

Class Bivalvia has diversified over the past 500 million years in a roughly exponential pattern [3] (figure 1). Both the infauna and epifauna followed this upward diversification trajectory, although infauna outpaced the epifauna starting in the Jurassic, ultimately becoming three times as rich by the Miocene (figure 1*a,b*; electronic supplementary material, table S2). Each group shows high temporal variation in diversification rate, but the average over the Phanerozoic barely exceeds nil in either group (figure 1*c,d*), a nice illustration of the compounding effect of even a slightly positive diversification rate over hundreds of millions of years.

Mass extinctions depleted diversity in both groups, disproportionately affecting infauna at the end-Permian and epifauna at the end-Cretaceous, but both groups re-diversified after these and other events [3] (figure 1*a,b*). Analysing and interpreting each fluctuation in these diversity curves is beside our main objective, but certain patterns observed here align with prior knowledge of bivalve diversification. For example, the relatively high standing diversity of the epifauna in the Late Cretaceous reflects the exceptional diversification of the tropical-platform rudists and the reclining gryphaeids and inoceramids.

Across the study interval, infaunal and epifaunal genera, and bivalves as a whole, show that extinction rates do not increase with diversity (figure 2*i–l*; table 1), consistent with previous analyses [7,57,58]. These distinct ecological groups, however, show different dynamics in their rates of origination and net diversification. Infaunal genera have experienced relatively weak, and statistically insignificant, diversity-dependence in origination rate and net diversification rate (figure 2*a,b,e,f*; table 1). Epifauna, by contrast, show stronger and statistically significant diversity-dependence in both their origination and net diversification rates (figure 2*c,d,g,h*; table 1). Rather than rebounding only from major mass extinctions, origination and net diversification of the epifauna were both enhanced when diversity was reduced and suppressed when diversity increased.

Patterns of diversity-dependence in origination and net diversification rates for both life modes are largely insensitive to variations in analytical protocol (electronic supplementary material, table S4). Starting the study interval after the initial major pulse of diversification in the Early Ordovician, or, even more extreme, after the entire Ordovician radiation, did not impact the observed diversity-dependence in evolutionary rates. Likewise, results persist, albeit somewhat muted, if the immediate aftermaths of major mass extinctions are excluded. Results were also robust to details of detrending diversity and evolutionary rates, and to the exclusion of genera lacking finely resolved (mainly substage) first and last appearances (e.g. last appearance as ‘Maastrichtian’ instead of ‘Upper Maastrichtian’) (electronic supplementary material, tables S1, S2). Simulations that randomly assign genera to two groups show that the difference in diversity-dependence between these randomized groups rarely exceeds the observed infaunal–epifaunal difference (3% of randomizations for origination; 1% for net diversification) (electronic supplementary material, figure S3). Thus, stronger diversity-dependence in epifauna versus infauna is a robust feature that is unlikely to have arisen by chance, and we therefore conclude that the difference in their diversity dynamics is meaningful and potentially interpretable biologically.

Evolutionary rates of the two groups within the same time interval are strongly and positively correlated (table 2), consistent with their similar, roughly exponential, diversification histories (figure 1*a–d*). The correlation is not perfect, however, and the residual variation must account for the observed difference in diversity dynamics between the two groups. Comparing rates with a temporal lag, diversification rate in either group fails to predict the rate in the other group in the following time interval, contrary to what we would expect if one group were suppressing diversification in the other. This limited analysis suggests that there is no prima facie evidence for negative interaction between the two groups at this temporal scale.

**Table 2.** Cross-correlations of net diversification rate.

analysis	product–moment correlation	*p*-value
infaunal versus epifaunal, lag 0	0.84	< 0.001
infaunal leading epifaunal, lag 1	0.014	0.89
epifaunal leading infaunal, lag 1	−0.016	0.88

### (b) Diversification despite diversity-dependence

Negative diversity-dependence in bivalve evolution despite their steady Phanerozoic diversification suggests that feedbacks have operated throughout their history, without setting a ceiling on their total diversity. This result supports the view that diversity-dependence can operate in a macroevolutionary system even when the overall trajectory of that system is positive rather than asymptotic [3,7,53]. It also stands in contrast to most modelling of macroevolutionary systems, whether based on palaeobiological data or evolutionary trees of living species, that assumes systems are near a fixed carrying capacity for much of their history [2,11,52,59]. One long-standing hypothesis is that background and mass extinction intervene before a theoretical accommodation limit is reached [60,61]; in addition, increased nutrient inputs through the Phanerozoic may have raised diversity limits on the long term [62,63].

The overall bivalve pattern in diversity-dependence is primarily driven by one life mode, the epifauna, as predicted. By contrast, the short-term ecological interactions among infaunal bivalves [31,32] do not manifest at macroevolutionary scales. As observed in other systems [7,60,61,64–67], negative diversity-dependence operates via origination, not extinction. However, diversity-dependent control of diversification by damping or promoting origination has been difficult to explain [10,57]. The most frequent hypothesis involves variations in the survival of incipient species [57,68–70]. Such incipient species typically have small population sizes and narrow geographical ranges, so that temporal variation in biotic and abiotic pressures can alter the probability of establishment as discrete taxa detectable in the fossil record [70,71]. This effect may be strongest among epifauna given their more limited habitat space and greater exposure to physical and biotic disturbances, pressures that would be intensified or relaxed under times of positive or negative diversity excursions, respectively. Although predation intensity has increased over the past 500 million years [30], this trend does not dominate our results; rather, negative diversity-dependence is evident throughout the Phanerozoic (figure 2, electronic supplementary material, figure S2).

The ecological difference we find in diversity-dependence is unlikely simply to reflect clade-specific differences, because infaunal versus epifaunal life mode transcends clade membership (electronic supplementary material, table S2). The primitive life mode for Cambrian bivalves remains uncertain [72], but no matter how that controversy is resolved, the two modes of life are polyphyletic within and among taxonomic orders; nine of the 25 bivalve orders contain both infaunal and epifaunal taxa, signifying at least nine transitions. Thus, epifaunality is likely a deterministic factor in diversity dynamics, given its multiple, independent derivations across distantly related clades and their broad variety of life histories and basic body plans.

Throughout their evolutionary history, bivalve diversification has entailed both functional novelty and continued subdivision of those functions [73,74]. However, epifauna have attained less functional variety than have the infauna [23], which may help to explain both the lower standing diversity of the group today and the stronger diversity-dependence in its origination rate throughout the Phanerozoic. Thus, for epifauna, short-term runs above the long-term diversity trend might reflect the rare acquisition of new functions and their subdivision, briefly allowing relatively unimpeded diversification that is later damped by negative feedbacks. The infauna may show a steadier establishment of new functions and minor differentiation within them, thereby reducing the kind of interference that would impose significant diversity dependence. Alternatively, enhanced origination owing to abiotic factors such as continental flooding and greater provinciality [75–77], might promote the evolution of new functions, which themselves persist when the promoting conditions fade. Testing these scenarios would require determining whether excursions above the long-term diversity trend were mostly initiated by the evolution of entirely new functions; whether taxonomic diversification, by sheer numbers, itself promoted the evolution of new functions; or whether diversification reflected a proliferation of lineages by finer subdivision of existing functions [78]. Considering the enormous population sizes of many bivalve species, it seems unlikely that such subdivision of niches has reached the theoretical limit set by the smallest sustainable biomass within each species [79]. High-dimensional functional groups of genera (i.e. combinations of motility, tiering, attachment, and feeding) have not been comprehensively assigned to all genera at sufficient temporal resolution [23,73,74]; thus, we cannot yet fully analyse the evolutionary sequence of niche subdivision and the effects of niche packing on diversity dynamics [80].

### (c) Coda

Diversity-dependence on macroevolutionary scales remains a difficult but engaging problem, and the stakes are high for determining proximate and ultimate causes given the environmental and climatic turmoil now challenging today's biodiversity [81]. Fossil data have long shown that diversification dynamics in response to episodes of major environmental and climatic change can yield accelerated rebounds in biodiversity following catastrophic losses during mass extinctions. Although these rebounds eventually slow down [3,67], diversity continues to rise, posing the fundamental question of how geologically short-term diversity-dependence within a biological group can be reconciled with its long-term diversity accumulation. This phenomenon is not fully understood, but it is now clearer that a complete theory must involve enhanced diversification at times of reduced diversity; suppressed diversification at times of higher diversity; the asymmetry between origination and extinction; the ability of diversity to ‘overshoot’ the long-term trend; and the reasons for the long-term trend itself. Partitioning a major clade into two ecologically defined groups of potentially interacting lineages, we find diversity-dependence in just one, identifying a new set of testable hypotheses for the factors underlying the striking result that diversity-dependence can operate even as the clade and its two ecological groups continue to diversify.

## Data Availability

All data and code are uploaded as electronic supplementary material [82].

## References

[1] Rillo MC, Etienne RS. 2022 Diversity-dependent diversification. Oxford Bibliogr. Evol. Biol. **10**, 9780199941728. (10.1093/OBO/9780199941728-0141)

[2] Sepkoski Jr JJ. 1984 A kinetic model of Phanerozoic taxonomic diversity. III. Post-Paleozoic families and mass extinctions. Paleobiology **10**, 246-267. (10.1017/S0094837300008186)

[3] Miller AI, Sepkoski Jr JJ. 1988 Modeling bivalve diversification: the effect of interaction on a macroevolutionary system. Paleobiology **14**, 364-369. (10.1017/S0094837300012100) 11542146

[4] Sepkoski Jr JJ, McKinney FK, Lidgard S. 2000 Competitive displacement among post-Paleozoic cyclostome and cheilostome bryozoans. Paleobiology **26**, 7-18. (10.1666/0094-8373(2000)026<0007:CDAPPC>2.0.CO;2) 11543303

[5] Ezard THG, Aze T, Pearson PN, Purvis A. 2011 Interplay between changing climate and species' ecology drives macroevolutionary dynamics. Science **332**, 349-351. (10.1126/science.1203060) 21493859

[6] Foote M, Cooper RA, Crampton JS, Sadler PM. 2018 Diversity-dependent evolutionary rates in early Palaeozoic zooplankton. Proc. R. Soc. B **285**, 20180122. (10.1098/rspb.2018.0122) PMC5832717 29491177

[7] Foote M. 2023 Diversity-dependent diversification in the history of marine animals. Am. Nat. **201**, 680-693. (10.1086/723626) 37130233

[8] Rabosky DL, Hurlbert AH. 2015 Species richness at continental scales is dominated by ecological limits. Am. Nat. **185**, 572-583. (10.1086/680850) 25905501

[9] Harmon LJ, Harrison S. 2015 Species diversity is dynamic and unbounded at local and continental scales. Am. Nat. **185**, 584-593. (10.1086/680859) 25905502

[10] Moen D, Morlon H. 2014 Why does diversification slow down? Trends Ecol. Evol. **29**, 190-197. (10.1016/j.tree.2014.01.010) 24612774

[11] Rabosky DL, Lovette IJ. 2008 Explosive evolutionary radiations: decreasing speciation or increasing extinction through time? Evolution **62**, 1866-1875. (10.1111/j.1558-5646.2008.00409.x) 18452577

[12] Quental TB, Marshall CR. 2009 Extinction during evolutionary radiations: reconciling the fossil record with molecular phylogenies. Evolution **63**, 3158-3167. (10.1111/j.1558-5646.2009.00794.x) 19659595

[13] Jackson JBC. 1977 Competition on marine hard substrata: the adaptive significance of solitary and colonial strategies. Am. Nat. **111**, 743-767. (10.1086/283203)

[14] Paine RT. 1984 Ecological determinism in the competition for space. Ecology **65**, 1339-1348. (10.2307/1939114)

[15] Stanley SM, Newman WA. 1980 Competitive exclusion in evolutionary time: the case of the acorn barnacles. Paleobiology **6**, 173-183. (10.1017/S0094837300006734)

[16] McKinney FK. 1995 One hundred million years of competitive interactions between bryozoan clades: asymmetrical but not escalating. Biol. J. Linn. Soc. **56**, 465-481. (10.1111/j.1095-8312.1995.tb01105.x)

[17] Lidgard S, Di Martino E, Zágoršek K, Liow LH. 2021 When fossil clades ‘compete’: local dominance, global diversification dynamics and causation. Proc. R. Soc. B **288**, 20211632. (10.1098/rspb.2021.1632) PMC8456135 34547910

[18] Jablonski D, Roy K, Valentine JW. 2006 Out of the tropics: evolutionary dynamics of the latitudinal diversity gradient. Science **314**, 102-106. (10.1126/science.1130880) 17023653

[19] Krug AZ, Jablonski D. 2010 Long-term origination rates are reset only at mass extinctions. Geology **40**, 731-734. (10.1130/G33091.1)

[20] Crouch NMA, Edie SM, Collins KS, Bieler R, Jablonski D. 2021 Calibrating phylogenies assuming bifurcation or budding alters inferred macroevolutionary dynamics in a densely sampled phylogeny of bivalve families. Proc. R. Soc. B **288**, 20212178. (10.1098/rspb.2021.2178) PMC8634622 34847770

[21] Stanley SM. 1970 Relation of shell form to life habits of the Bivalvia (Mollusca). Geol. Soc. Amer. Mem **125**, 1-296.

[22] Stanley SM. 1972 Functional morphology and evolution of bysally attached bivalve mollusks. J. Paleontol. **46**, 165-212.

[23] Edie SM, Jablonski D, Valentine JW. 2018 Contrasting responses of functional diversity to major losses in taxonomic diversity. Proc. Natl Acad. Sci. USA **115**, 732-737. (10.1073/pnas.1717636115) 29305556 PMC5789943

[24] Stanley SM. 1968 Post-Paleozoic radiation of infaunal bivalve molluscs: a consequence of mantle fusion and siphon formation. J. Paleontol. **42**, 214-229.

[25] Savazzi E. 1999 Boring, nestling, and tube-dwelling in bivalves. In Functional morphology of the invertebrate skeleton (ed. E Savazzi), pp. 205-277. New York, NY: John Wiley and Sons.

[26] Seilacher A, Gishlick AD. 2015 Morphodynamics. Boca Raton, FL: CRC Press.

[27] Thayer CW. 1983 Sediment-mediated biological disturbance and the evolution of marine benthos. In Biotic interactions in recent and fossil benthic communities (eds MJS Tevesz, PJ McCall), pp. 479-625. New York, NY: Plenum.

[28] Droser ML, Bottjer DJ. 1993 Trends and patterns of Phanerozoic ichnofabric. Annu. Rev. Earth. Planet. Sci. **21**, 205-225. (10.1146/annurev.ea.21.050193.001225)

[29] Miller AI. 1998 Biotic transitions in global marine diversity. Science **281**, 1157-1160. (10.1126/science.281.5380.1157) 9716540

[30] Vermeij GJ. 1987 Evolution and escalation. Princeton, NJ: Princeton University Press.

[31] Peterson CH, Andre SV. 1980 An experimental analysis of interspecific competition among marine filter feeders in a soft-sediment environment. Ecology **61**, 129-139. (10.2307/1937163)

[32] Wilson WH. 1990 Competition and predation in marine soft-sediment communities. Annu. Rev. Ecol. Syst. **21**, 221-241. (10.1146/annurev.es.21.110190.001253)

[33] Erwin DH. 2008 Macroevolution of ecosystem engineering, niche construction and diversity. Trends Ecol. Evol. **23**, 304-310. (10.1016/j.tree.2008.01.013) 18457902

[34] Kidwell SM, Jablonski D. 1983 Taphonomic feedback: ecological consequences of shell accumulation. In Biotic interactions in recent and fossil benthic communities (eds MJS Tevesz, PJ McCall), pp. 195-248. New York, NY: Plenum.

[35] Gutiérrez JL, Jones CG, Strayer SL, Iribarne OO. 2003 Mollusks as ecosystem engineers: the role of shell production in aquatic habitats. Oikos **101**, 79-90. (10.1034/j.1600-0706.2003.12322.x)

[36] Sepkoski Jr JJ. 2002 A compendium of fossil marine animal genera. Bulls Amer. Paleontol. **363**, 1-560.

[37] Jablonski D, Roy K, Valentine JW, Price RM, Anderson PS. 2003 The impact of the pull of the recent on the history of marine diversity. Science **300**, 1133-1135. (10.1126/science.1083246) 12750517

[38] Gradstein FM, Ogg JG, Schmitz MD, Ogg GM. 2012 The geologic time scale 2012. Amsterdam, The Netherlands: Elsevier.

[39] Gradstein FM, Ogg JG, Schmitz MD, Ogg GM. 2020 Geologic time scale 2020. Amsterdam, The Netherlands: Elsevier.

[40] Foote M. 2000 Origination and extinction components of taxonomic diversity: general problems. Paleobiology **26**, 74-102. (10.1666/0094-8373(2000)26[74:OAECOT]2.0.CO;2)

[41] Alroy J. 2014 Accurate and precise measures of origination and extinction rates. Paleobiology **40**, 374-397. (10.1666/13036)

[42] Warnock RCM, Heath TA, Stadler T. 2020 Assessing the impact of incomplete species sampling on estimates of speciation and extinction rates. Paleobiology **46**, 137-157. (10.1017/pab.2020.12)

[43] Foote M, Raup DM. 1996 Fossil preservation and the stratigraphic ranges of taxa. Paleobiology **22**, 121-140. (10.1017/S0094837300016134) 11539203

[44] Foote M, Sadler PM, Cooper RA, Crampton JS. 2019 Completeness of the known graptoloid palaeontological record. J. Geol. Soc. London **176**, 1038-1055. (10.1144/jgs2019-061)

[45] Raup DM. 1985 Mathematical models of cladogenesis. Paleobiology **11**, 42-52. (10.1017/S0094837300011386)

[46] Nee S. 2006 Birth–death models in macroevolution. Annu. Rev. Ecol. Evol. Syst. **37**, 1-17. (10.1146/annurev.ecolsys.37.091305.110035)

[47] Raup DM. 1978 Cohort analysis of generic survivorship. Paleobiology **4**, 1-15. (10.1017/S0094837300005649)

[48] Foote M, Sepkoski Jr JJ. 1999 Absolute measures of the completeness of the fossil record. Nature **398**, 415-417. (10.1038/18872) 11536900

[49] Foote M. 2022 Data from: Diversity-dependent diversification in the history of marine animals. Dryad Digital Repository. (10.5061/dryad.02v6wwq6d) 37130233

[50] Kidwell SM. 2005 Shell composition has no net impact on large-scale evolutionary patterns in mollusks. Science **307**, 914-917. (10.1126/science.1106654) 15705849

[51] Foote M. 2010 The geological history of biodiversity. In Evolution since Darwin (eds MA Bell, DJ Futuyma, WF Eanes, JS Levinton), pp. 479-510. Sunderland, MA: Sinauer.

[52] Sepkoski Jr JJ. 1978 A kinetic model of Phanerozoic taxonomic diversity. I. Analysis of marine orders. Paleobiology **4**, 223-251. (10.1017/S0094837300005972)

[53] Marshall CR, Quental TB. 2016 The uncertain role of diversity dependence in species diversification and the need to incorporate time-varying carrying capacities. Phil. Trans. R. Soc. B **371**, 20150217. (10.1098/rstb.2015.0217) 26977059 PMC4810812

[54] Kelly C, Price TD. 2005 Correcting for regression to the mean in behavior and ecology. Am. Nat. **166**, 700-707. (10.1086/497402) 16475086

[55] Alroy J. 2008 Dynamics of origination and extinction in the marine fossil record. Proc. Natl Acad. Sci. USA **105**, 11 535-11 542. (10.1073/pnas.0802597105) 18695240 PMC2556405

[56] R Core Team. 2023 R: a language and environment for statistical computing, version 4.3.0. Vienna, Austria: R Foundation for Statistical Computing.

[57] Rineau V, Smyčka J, Storch D. 2022 Diversity dependence is a ubiquitous phenomenon across Phanerozoic oceans. Sci. Adv. **8**, eadd9620. (10.1126/sciadv.add9620) 36306361 PMC9616491

[58] Guo Z, Flannery-Sutherland JT, Benton MJ, Chen Z-Q. 2023 Bayesian analyses indicate bivalves did not drive the downfall of brachiopods following the Permian–Triassic mass extinction. Nat. Commun. **14**, 5566. (10.1038/s41467-023-41358-8) 37689772 PMC10492784

[59] Rabosky DL. 2013 Diversity-dependence, ecological speciation, and the role of competition in macroevolution. Annu. Rev. Ecol. Evol. Syst. **44**, 481-502. (10.1146/annurev-ecolsys-110512-135800)

[60] Walker TD, Valentine JW. 1984 Equilibrium models of evolutionary species diversity and the number of empty niches. Am. Nat. **124**, 887-899. (10.1086/284322)

[61] Stanley SM. 2007 An analysis of the history of marine animal diversity. Paleobiology **33**, 1-55.

[62] Bambach RK. 1993 Seafood through time: changes in biomass, energetics, and productivity in the marine ecosystem. Paleobiology **19**, 372-397. (10.1017/S0094837300000336)

[63] Allmon WD, Martin RE. 2014 Seafood through time revisited: the Phanerozoic increase in marine trophic resources and its macroevolutionary consequences. Paleobiology **40**, 256-287. (10.1666/13065)

[64] Gilinsky NL, Bambach RK. 1987 Asymmetrical patterns of origination and extinction in higher taxa. Paleobiology **13**, 427-445. (10.1017/S0094837300009027)

[65] Raup DM, Boyajian GE. 1988 Patterns of generic extinction in the fossil record. Paleobiology **14**, 109-125. (10.1017/S0094837300011866) 11542145

[66] Stanley SM. 1990 Delayed recovery and the spacing of major extinctions. Paleobiology **16**, 401-414. (10.1017/S0094837300010150)

[67] Sepkoski Jr JJ. 1998 Rates of speciation in the fossil record. Philos. Trans. R. Soc. B **353**, 315-326. (10.1098/rstb.1998.0212) PMC1692211 11541734

[68] Stanley SM. 1986 Population size, extinction, and speciation: the fission effect in Neogene Bivalvia. Paleobiology **12**, 89-110. (10.1017/S0094837300003006)

[69] Allmon WD. 1992 A causal analysis of stages in allopatric speciation. Oxford Surveys in Evolutionary Biology **8**, 219-257.

[70] Rosenblum EB, Sarver BAJ, Brown JW, Des Roches S, Hardwick KM, Hether TD, Eastman JM, Pennell MW, Harmon LJ. 2012 Goldilocks meets Santa Rosalia: an ephemeral speciation model explains patterns of diversification across time scales. Evol. Biol. **39**, 255-261. (10.1007/s11692-012-9171-x) 22707806 PMC3364415

[71] Crampton JS, Cooper RA, Foote M, Sadler PM. 2020 Ephemeral species in the fossil record? Synchronous coupling of macroevolutionary dynamics in mid-Paleozoic zooplankton. Paleobiology **46**, 123-135. (10.1017/pab.2020.3)

[72] Stanley SM. 2015 Treatise Online no. 71: Part N, Revised, Volume 1, Chapter 5: Functional morphology of noncementing Bivalvia. *Treatise Online.* (10.17161/to.v0i0.5054)

[73] Mondal S, Harries PJ. 2016 Phanerozoic trends in ecospace utilization: the bivalve perspective. Earth Sci. Rev. **152**, 106-118. (10.1016/j.earscirev.2015.10.005)

[74] Zhou S, Edie SM, Collins KS, Crouch NMA, Jablonski D. 2023 Cambrian origin but no early burst in functional disparity for class Bivalvia. Biol. Lett. **19**, 20230157. (10.1098/rsbl.2023.0157) 37254520 PMC10230185

[75] Valentine JW. 1973 Evolutionary paleoecology of the marine biosphere. Englewood Cliffs, NJ: Prentice-Hall.

[76] Zaffos A, Finnegan S, Peters SE. 2017 Plate tectonic regulation of global marine animal diversity. Proc. Natl Acad. Sci. USA **114**, 5653-5658. (10.1073/pnas.1702297114) 28507147 PMC5465924

[77] Holland SM. 2018 Diversity and tectonics: predictions from neutral theory. Paleobiology **44**, 219-236. (10.1017/pab.2018.2)

[78] Valentine JW. 1969 Patterns of taxonomic and ecological structure of the shelf benthos during Phanerozoic time. Palaeontology **12**, 684-709.

[79] Levinton JS. 1979 A theory of diversity equilibrium and morphological evolution. Science **204**, 335-336. (10.1126/science.204.4390.335) 17800361

[80] Wagner PJ. 2000 Exhaustion of morphological character states among fossil taxa. Evolution **54**, 365-386. 10937214 10.1111/j.0014-3820.2000.tb00040.x

[81] Jablonski D, Edie SM. 2023 Perfect storms shape biodiversity in time and space. Evol. J. Linn. Soc. Lond. **2**, kzad003. (10.1093/evolinnean/kzad003)

[82] Foote M, Edie SM, Jablonski D. 2024 Ecological structure of diversity-dependent diversification in Phanerozoic marine bivalves. Figshare. (10.6084/m9.figshare.c.6996723) PMC10792395 38229556

